# Bad vessels beware! Semaphorins will sort you out!

**DOI:** 10.15252/emmm.201505551

**Published:** 2015-08-26

**Authors:** Guido Serini, Luca Tamagnone

**Affiliations:** Department of Oncology, University of Torino and Candiolo Cancer Institute, FPO – IRCCSCandiolo, Italy

## Abstract

Secreted class 3 semaphorins (Sema3), which signal through plexin receptors and mostly use neuropilins (Nrps) as co-receptors, were initially identified for their ability to steer navigating axons in the developing embryo. They were later found to control angiogenesis in physiological and pathological settings as well (Serini *et al*, 2013). Indeed, the development of a novel and aberrant vasculature is central to the pathogenesis of several human diseases, including cancer and vascular retinopathies (Goel *et al*, 2011). A large body of evidence demonstrates that in cancer, a massive regression of angiogenesis may trigger hypoxia-driven genetic programs, which in turn can overcome drug inhibitory mechanisms and ultimately favour cancer cell invasion and dissemination. Thus, an emerging concept in molecular medicine is to devise therapeutic strategies that, rather than simply inhibiting angiogenesis, can foster the re-establishment of a structural and functional normal network, a phenomenon often referred to as “vessel normalization” (Goel *et al*, 2011) (Fig 1). Of note, and in this context, Sema3A (Maione *et al*, 2009) and Sema3F (Wong *et al*, 2012) have been reported to favour the normalization of cancer vasculature and impair metastatic dissemination.

See also: **W-J Yang *et al*** (October 2015)

Although Sema3C shares many molecular features with the other Sema3 family members, unlike other secreted semaphorins that are mainly found to act as inhibitors or repulsive factors, Sema3C appears to have an attracting role in axon guidance (Sanyas *et al*, 2012) and promotes cell migration during embryo development (Epstein *et al*, 2015). Both members of the Nrp family, Nrp1 and Nrp2, can bind Sema3C, but their functional role in the signalling cascade is unclear. Notably, Sema3C has never been found to bind plexins directly; however, both PlexinA2 and PlexinD1 have been implicated in Sema3C signalling, based on genetic evidence in developmental models (Epstein *et al*, 2015). The work of Yang and collaborators published in this issue of *EMBO Molecular Medicine* provides the first description of the molecular mechanisms by which Sema3C can impact on pathological blood vessel formation, clearly indicating an inhibitory activity in this context (Yang *et al*, 2015). Moreover, this study proposes potential therapeutic applications of Sema3C in counteracting pathological angiogenesis by targeting immature vessel sprouts without affecting quiescent established vascular networks (Fig 1). These data are consistent with another recent study analysing the role of Sema3C in lymphatic endothelial cells (LECs) and the vascular network in tumour progression (Mumblat *et al*, 2015).

**Figure 1:**
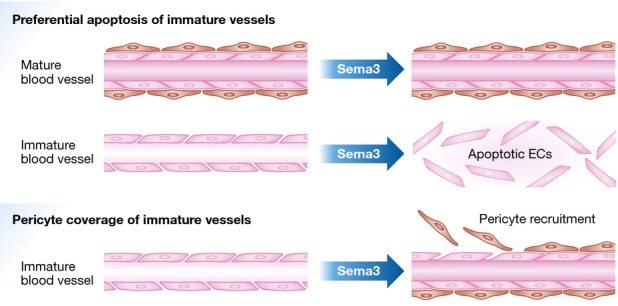
Sema3 proteins display vascular normalizing properties Secreted Sema3A (Maione *et al*, 2009), Sema3C (Yang *et al*, 2015), and Sema3F (Wong *et al*, 2012) promote the functional normalization of pathological blood vessels that are devoid of pericyte ensheathment. Of note, both Sema3A (Maione *et al*, 2009) and Sema3C (Yang *et al*, 2015) appear to selectively elicit the apoptosis EC belonging to immature, but not mature blood vessels (upper panel). In addition, Sema3A has chemoattractive activity towards cultured SMCs (Maione *et al*, 2009), supporting the hypothesis that at least this Sema3 may also favour the recruitment of pericytes on immature blood vessels (lower panel).

Yang and colleagues employed a mouse model of oxygen-induced retinopathy (OIR) that recapitulates the disease observed in prematurely born babies, whereby localized hypoxia drives the formation of intravitreal neovascular tufts. Of note, secreted semaphorins (including Sema3A and Sema3C) are present in the retina during this process, but their inhibitory activity cannot prevent new vessel sprouting into the vitreous, potentially leading to blindness. The authors found that intravitreal administration of recombinant Sema3C significantly inhibited pathologic angiogenesis in the OIR model. This is in accord with the previous findings on Sema3A, suggesting that these two molecules, while often displaying divergent functions in development, behave similarly in angiogenesis.

Sema3C, secreted by both neural crest cells and the derived smooth muscle cells (SMCs), was also recently shown to regulate vascular ECs in a paracrine fashion to control heart outflow tract septation (Plein *et al*, 2015). Consistently, the model elaborated by Yang and colleagues implies that Sema3C, released by SMCs and pericytes, acts locally on adjacent ECs to counteract and balance VEGF-induced angiogenesis. This inhibitory mechanism seems to be specific for immature vessels, possibly because of the selective expression of the implicated receptors, or simply due to higher susceptibility to plexin-dependent signals inhibiting cell-substrate adhesion and cytoskeletal dynamics. The latter would imply a constraint mechanism on vessel sprouting, which would be allegedly overcome in the presence of strong pro-angiogenic signals. In their study, Yang and co-workers demonstrated that a high dose of Sema3C in the microenvironment could bolster this restraint, resulting in selective pruning of immature vessels (Yang *et al*, 2015). This mechanism may not be merely based on quantitative balancing of pro-angiogenic and anti-angiogenic signals, but could imply a vessel-type-specific combination of Sema3C receptor components. For instance, Yang *et al* (2015) show that Sema3C inhibitory activity on ECs depends on the expression of Nrp1 and PlexinD1 that, being enriched in immature new vessel sprouts, make them especially sensitive to Sema3C-dependent inhibition. In principle, Sema3C (or specific PlexinD1 ligands, such as Sema3E; Casazza *et al*, 2012) could therefore be used to target neo-angiogenic processes without affecting the normal vasculature. Notably, according to Mumblat *et al* (2015), Sema3C inhibitory activity on LECs depends on both PlexinD1 and PlexinA1. Moreover, exogenous Sema3A, which is thought to signal in ECs via Nrp1 and PlexinA1/A4, was also found to trigger the apoptosis of aberrant and immature blood vessels in cancer (Maione *et al*, 2009); intriguingly, the data suggested that Sema3A might promote vessel normalization also by recruiting SMCs (Fig 1), a mechanism that was not observed by Yang *et al* (2015) for Sema3C. It is conceivable that, depending on the EC receptor expression repertoire, different semaphorins may prove therapeutically viable tools to selectively prune immature and abnormal blood vessels in specific pathologies, while sparing mature and normal vasculature. In this respect, further investigation of the specific expression pattern of semaphorin receptors in different functional stages of vessels and ECs would prove highly valuable.

Yang and co-workers found significant induction of blood vessel EC apoptosis by Sema3C, while Mumblat *et al* (2015) observed reduced LEC proliferation; VEGF signalling was partly impaired, but the implicated mechanisms were not elucidated. In both studies, Sema3C treatment elicited rapid actin cytoskeleton remodelling and loss of cell-substrate adhesion; moreover, Yang *et al* (2015) report that, in response to Sema3C, ECs undergo fast remodelling of cell–cell junctions as well. The observed phenotype recalls the typical cellular “collapse” or contraction, well described in response to other semaphorins (Barberis *et al*, 2004). It can be postulated that ECs that are unable to efficiently adhere to the extracellular matrix or form stabilizing cell junctions, especially if they lack support from mural cells in immature vessels, are then primed to enter apoptosis. Notably, the Tufro laboratory had reported instead that Sema3C promotes glomerular EC adhesion, migration, and proliferation (Banu *et al*, 2006). The possibility remains therefore that ECs from different vascular beds might display divergent biological responses to Sema3C, possibly due to the different expression profile of cell surface receptors.

Another mechanistic aspect investigated by Yang *et al* (2015) is the regulatory role of proteolytic cleavage in Sema3C signalling (Fig 2). Similar to other secreted semaphorins, Sema3C is the target of furin family proteases, which can detach the sema and PSI domains from the C-terminal portion known to bind Nrp1, which is a key component of the receptor complex. Indeed, furin cleavage was found to blunt inhibitory signals mediated by all semaphorins that depend on Nrps, including Sema3C (as confirmed in this study). By analogy to Sema3A and Sema3B, this increases the interest in engineering furin-resistant Sema3C variants with increased functional stability, for therapeutic applications. Of note, one could envision that the immature-vessel-specific activity of the cleavable Sema3C used in this study may be influenced by its capacity to elicit a strong, but short-lived inhibitory signalling in ECs.

**Figure 2:**
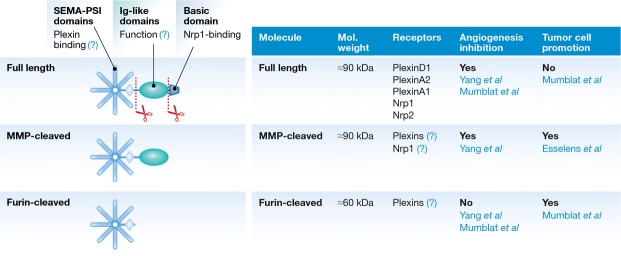
Sema3C activity is regulated by proteolytic cleavage Sema3C is synthesized as a secreted full-length molecule, with a basic-charged C'-tail putatively tethering it to the cell surface and interacting with the co-receptor Nrp1. MMPs have been found to remove this C'-sequence, enhancing Sema3C extracellular diffusion. On the other hand, furin-like convertases are found to release the sema domain from the rest of the molecule; this ablates the inhibitory activity for EC and vessels, but allegedly preserves other functions, such as tumour promotion.

Sema3C is also the target of metalloproteases (MMPs), which only release a very short C-terminal sequence (Esselens *et al*, 2010); while not significantly affecting protein structure, this event removes the main Nrp1-binding sequence. According to Yang and co-workers, unlike furin-processed Sema3C, MMP-processed Sema3C is functionally competent for EC inhibition, which leaves open the question on the requirement for Nrp1 signalling in this pathway, and would suggest a currently unknown function of the Ig-like domain of Sema3C (Fig 2). Esselens and co-workers had shown that MMP-processed Sema3C promotes tumour cell migration (Esselens *et al*, 2010); furthermore, Lee and Yutzey (2011) showed that Sema3C expression is induced by Twist1, an epithelial to mesenchymal transition (EMT)-inducing transcription factor implicated in cancer progression. Finally, Mumblat *et al* (2015) showed that furin-cleaved Sema3C, while deficient for inhibitory activity on ECs, can promote cancer cell viability. These findings fit with the multiple reports indicating elevated expression of Sema3C in highly malignant human tumours and suggest the ability of tumour cells to switch Sema3C function from an anti-angiogenic to a growth-promoting one, by means of proteolytic processing. There is as yet little evidence suggesting an important role of proteolytic processing in attractive Sema3C functions in neural and cardiac development. Thus, the current scenario nominates Sema3C as a dual-function molecule, similar to Sema3E (which intriguingly also relies on PlexinD1 as major signalling receptor). Further studies will allow the elucidation of the different signalling cascades responsible for these apparently divergent functional activities of Sema3C.

In conclusion, while Sema3C has so far been mainly considered as an attractive cue for neurons and a promoter of developmental morphogenesis, the study of Yang and co-workers reveals how, in mouse models, recombinant Sema3C may effectively and selectively block hypoxia-driven aberrant angiogenesis, without affecting normal quiescent vessels. The data also suggest that differential expression of receptors in target cells and proteolytic maturation of Sema3C may switch between these apparently antagonistic functions. Further insights into these aspects will allow full understanding of the activity of this semaphorin, with an eye to its application in medicine for blocking pathological angiogenesis, such as in retinopathies and cancer.
